# Supplementary material for: Areas of Concern and Support among the Austrian General Population: A Qualitative Content Analytic Mapping of the Shift between Winter 2020/21 and Spring 2022
Source: Healthcare (Basel). 2023 Sep 14;11(18):2539. doi: 10.3390/healthcare11182539 (PMC10530781; doi:10.3390/healthcare11182539)
Supplement: Supplementary file 1 [file healthcare-11-02539-s001.zip › healthcare-2556043-supplementary.pdf]

Table S1: Category System that Emerged from the Data of Question 1: " What currently gives you the most cause for concern?"

Table S1. Category System that Emerged from the Date of Question 1: " What currently gives you the most cause for concern?"

Categories	*n*	%
**Inflation/Finances**	**294**	**30.4%**
Inflation	181	18.7%
Finances	113	11.7%
**War in Ukraine**	**212**	**21.9%**
**Mental health**	**101**	**10.5%**
Uncertainty/Anxiety	30	3.1%
Stress/Overburden	21	2.2%
Mental Health	14	1.4%
People	11	1.1%
Death of Relatives	8	0.8%
The Weather	7	0.7%
Loneliness	6	0.6%
Life/Everything	4	0.4%
**Physical health**	**101**	**10.5%**
Physical Health	81	8.4%
Disease in the Family	20	2.1%
**Pandemic**	**90**	**9.3%**
**Sociopolitical Development**	**88**	**9.1%**
Political and Social Developments	38	3.9%
Climate Change/Environmental Problems	22	2.3%
Situation in the World	16	1.7%
Media	8	0.8%
Refugees	4	0.4%
**Family/Relationship and Interpersonal Problems**	**80**	**8.3%**
Self-Related	42	4.3%
Relationship Problems	23	2.4%
Family Problems	16	1.7%
**Nothing**	**64**	**6.6%**
No Burden	58	6.0%
No Idea	6	0.6%
**Work/Unemployment**	**64**	**6.6%**
**School/Education/Study**	**38**	**3.9%**
**Restrictions**	**34**	**3.5%**

Table S2: Category System that Emerged from the Data of Question 2: "What currently provides you with the most support?"

Table S2. Category System that Emerged from the Date of Question 2: "What currently provides you with the most support?"

Categories	*n*	%
**Social Contacts**	**345**	**36.2%**
Family Support	145	15.2%
Friends/Colleagues/Classmates	82	8.6%
Conversation with Family/Friends	55	5.8%
Partner	39	4.1%
My Children/Grandchildren	16	1.7%
Time with Family/Friends	8	0.8%
**Recreational Activities**	**218**	**22.9%**
Leisure Activities	63	6.6%
Walking/Nature	51	5.4%
Sports	38	4.0%
Silence and Relaxation	32	3.4%
Music	16	1.7%
Hobbies	13	1.4%
Reading	5	0.5%
**Attitude and Abilities**	**209**	**21.9%**
Personal Attitude	68	7.1%
Positive Attitude	41	4.3%
Myself	29	3.0%
Faith	23	2.4%
Mental Skills	14	1.5%
Planning/Structuring	12	1.3%
Meditation	9	0.9%
Problem Solving	7	0.7%
Show Emotions	6	0.6%
**Nothing**	**109**	**11.4%**
Nothing	90	9.4%
Nobody/no Help	19	2.0%
**Distraction**	**85**	**8.9%**
Distraction	35	3.7%
Social Media	15	1.6%
Sleep	14	1.5%
Alcohol/Cigarettes	8	0.8%
Drugs	8	0.8%
Retreat	5	0.5%
**Work/Save Money**	**84**	**8.8%**
Work	39	4.1%
Save Money	33	3.5%
Money	12	1.3%
**Professional Help**	**42**	**4.4%**

Psychotherapy	21	2.2%
Medical Treatment	12	1.3%
Medication	9	0.9%
**Media and News**	**32**	**3.4%**
Reduce News	22	2.3%
News	10	1.0%
**I do not know**	27	2.8%
No Problem	16	1.7%
No Idea	11	1.2%
**Displacement**	**19**	**2.0%**
**Pets**	**11**	**1.2%**
**School and Education**	**9**	**0.9%**
**Others**	**9**	**0.9%**
